# Broadband Dielectric Response of Group-II Metal Oxide Monolayers: From Ionic to Electronic Polarization

**DOI:** 10.3390/mi17050564

**Published:** 2026-05-01

**Authors:** Pei Yin, Dongliang Jia, Dan Tan, Rusen Yang

**Affiliations:** 1 School of Physics, Xidian University, Xi’an 710126, China; 19141110581@stu.xidian.edu.cn; 2 School of Advanced Materials and Nanotechnology, Xidian University, Xi’an 710126, China; dliangjia@163.com

**Keywords:** Group-II metal oxide monolayers, ionic polarization, electronic polarization, low-frequency dielectric response, high-frequency dielectric response

## Abstract

The dielectric response provides an integral description of polarization mechanisms across frequency ranges and constitutes a key physical basis for understanding ferroelectric behavior. Here, we systematically investigate the broadband dielectric response of Group-II metal oxide (BeO, MgO, CaO, ZnO, and CdO) monolayers using first-principles calculation. In the low-frequency regime, ionic polarization governs the dielectric response. A distinctive feature is the LO–TO degeneracy at the Γ point accompanied by a V-shaped nonanalytic LO phonon dispersion. d-state hybridization increases with the metal atomic number, resulting in higher Born effective charge, which works together with phonon softening, reduced mass and unit cell area to significantly strengthen the ionic dielectric contribution. The quasiparticle band gap decreases with the metal atomic number, driving redshifts of the dielectric function and wide band optical response from the deep-ultraviolet to the near-infrared. Particularly, CdO exhibits the strongest electronic polarization, with an optical dielectric constant of 2.68 and a static refractive index of 1.64. This work establishes a complete dielectric spectrum from ionic to electronic polarization, providing theoretical guidance for polarization engineering and design of two-dimensional ferroelectric devices.

## 1. Introduction

Ferroelectric materials, with their switchable spontaneous polarization, show great potential for next-generation devices such as non-volatile memory, negative capacitance transistors, and neuromorphic computing devices [1,2]. The dielectric response is contributed from ionic polarization and electronic polarization, which dominate the low-frequency and the optical-frequency regime, respectively [3]. The polarization mechanisms in traditional perovskite ferroelectrics are well understood [4], and the discovery of sliding ferroelectricity in two-dimensional (2D) materials inspired increasing research efforts in monolayer materials. Although monolayer h-BN has no net spontaneous polarization, both simulations and experiments have shown that parallel stacking of h-BN in AB (or BA) registry breaks global inversion symmetry, and interlayer charge redistribution induces an out-of-plane polarization [5]. Meanwhile, the reduced dimensionality leads to weakened dielectric screening [6], resulting in fundamental differences in polarization responses between 2D and three-dimensional (3D) systems [7,8].

Group-II metal oxide (MO, M = Be, Mg, Ca, Zn, Cd) monolayers are isostructural to the h-BN monolayer and intrinsically lack inversion symmetry, making them promising building blocks for sliding ferroelectric architectures. Consequently, a comprehensive understanding of their broadband dielectric properties is critical to the rational design of associated ferroelectric devices. This family spans Group IIA (Be, Mg and Ca) and Group IIB (Zn and Cd), exhibiting diverse structures and electronic configurations and providing an ideal platform for investigating ionic and electronic polarization within a unified framework. Nevertheless, existing studies have investigated the dielectric and optical response of individual MO monolayers, such as the complex dielectric function of BeO, MgO, and CaO monolayers [9,10,11], the phonon spectrum of ZnO monolayer [12], and the optical response of BeO and CaO monolayers [10,13]. While these studies provided valuable insights into the dielectric or optical properties of individual MO monolayers, they addressed single materials and focused on narrow frequency ranges. A complete dielectric spectrum covering the evolution from low-frequency ionic polarization to high-frequency electronic polarization remains to be established across the entire Group-II MO family. Specifically, the key physical quantities, such as phonon dispersions, Born effective charges (BECs), the frequency-dependent dielectric function, and the optical response, remain to be elucidated.

Here, we utilize first-principles calculation to systematically study the broadband dielectric response of BeO, MgO, CaO, ZnO, and CdO monolayers. By analyzing phonon dispersions, BECs, complex dielectric function, optical absorption spectra and refractive index across this series, we establish the composition-dependent evolution of ionic and electronic polarization. The complete dielectric spectrum provides a physical framework for understanding polarization in 2D non-centrosymmetric materials and offers theoretical guidance for the future design of sliding ferroelectric devices via chemical tuning. We emphasize that this study is primarily concerned with the intrinsic dielectric properties of MO monolayers, rather than the ferroelectric switching in the sliding ferroelectric structure. The latter is a distinct phenomenon and left for future studies.

## 2. Computational Methods

All calculations were based on density functional theory with plane-wave basis sets and the projector augmented wave (PAW) method using the Vienna Ab initio Simulation Package (VASP) software [14]. The exchange–correlation effects were treated within the generalized gradient approximation using the Perdew–Burke–Ernzerhof functional, and the PAW method was used to describe the interaction between electrons and ions [15,16]. A plane-wave kinetic energy cutoff of 520 eV was used. In order to avoid the interaction between periodic images, we set a vacuum layer of 20 Å along the *z* direction. For structural relaxations of the MO monolayers, all atomic positions were optimized until the total energy change between successive ionic steps was below 10^−6^ eV and the maximum residual force on each atom was below 0.01 eV Å^−1^. Phonon dispersions were calculated using the finite-difference method within a 5 × 5 × 1 supercell. BECs were obtained from density functional perturbation theory (DFPT). To obtain the complex dielectric function and the corresponding optical properties, we performed Many-body perturbation theory calculations utilizing the *GW*_0_ approach combined with the Bethe–Salpeter equation (BSE), explicitly accounting for electron–electron and electron–hole interactions. The *GW*_0_ and BSE calculations employed a dielectric cutoff energy of 200 eV and 50 frequency points with a Lorentzian broadening of 0.01 eV applied to the optical spectra. During the calculation process, the NBANDS parameter was set to 120, which included 104 unoccupied bands. This was sufficient for conducting *GW*_0_ and BSE calculations using VASP. Other researchers also adopted a similar method of increasing the number of unoccupied bands to accurately calculate the complex dielectric function. The k-point meshes used throughout the calculation were as follows: 12 × 12 × 1 for structural relaxation, 18 × 18 × 1 for DFPT, 15 × 15 × 1 for the *GW*_0_ approximation, and 7 × 7 × 1 for solving BSE calculations. Post-processing was performed using the VASPKIT code [17].

## 3. Results and Discussion

### 3.1. Crystal Structure and Static Charge Distribution

**Figure 1:**
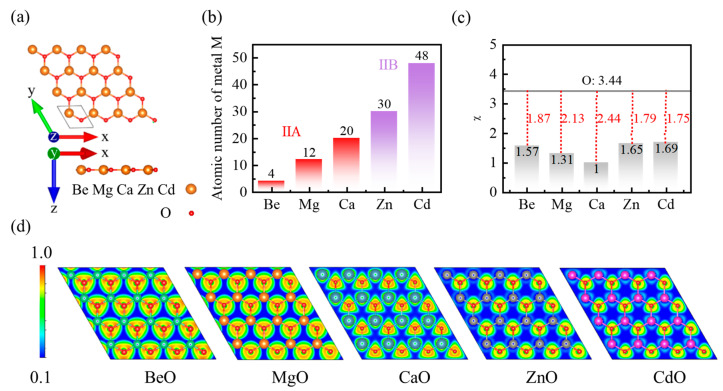
(**a**) Crystal structure, (**b**) atomic number of metal M (M = Be, Mg, Ca, Zn, Cd), (**c**) electronegativity difference, and (**d**) bonding characteristics of the MO monolayers.

As 2D materials isostructural to the h-BN monolayer, the MO monolayers provide a key platform for investigating sliding ferroelectrics. An assessment of ionic and electronic polarization in the dielectric response is a prerequisite for understanding ferroelectric materials from this class. Accordingly, we first present the crystal structures and static charge distributions of five MO monolayers, thereby establishing the basis for our subsequent analysis of polarization mechanisms. As shown in Figure 1a, M and O atoms are arranged in an alternating pattern, forming a non-centrosymmetric hexagonal honeycomb lattice (space group $P\bar{6}m2$). The absence of inversion symmetry is the structural origin of their intrinsic polar nature. After full structural relaxation, the lattice constants *a* are 2.68, 3.30, 3.78, 3.29 and 3.68 Å, and the in-plane bond lengths *d* are 1.55, 1.91, 2.18, 1.90 and 2.13 Å, respectively. These values agree well with previous reports [9,11,18,19,20]; a detailed comparison of the lattice constants is provided in Table S1. With increasing metal atomic number (Figure 1b), both *a* and *d* increase monotonically within each chemical group. The choice of these five materials follows a comparative design. Since Be and Mg have no occupied d electrons, the dielectric response of the BeO and MgO monolayers predominantly stems from intrinsic elemental properties like atomic radius and electronegativity, serving as a baseline control group that effectively isolates the d-orbital effects. Although Ca is also a Group IIA element without occupied d electrons, its unoccupied 3d states are relatively low-lying, providing a key opportunity to investigate possible hybridization involving empty d orbitals and its effect on the dielectric response. By contrast, the Group IIB elements Zn and Cd, while having lattice constants and bond lengths comparable to those of Mg and Ca, possess a filled d^10^ configuration, introducing pronounced d-electron effects. This is expected to substantially modify the orbital hybridization with O atoms and the electron-density distribution, thereby affecting the dielectric response.

Electronegativity difference between constituent atoms is an important driving force for charge transfer. As shown in Figure 1c, the O atom has a high electronegativity (χ = 3.44, Pauling scale), while the electronegativities of the M atoms are Be: 1.57, Mg: 1.31, Ca: 1.00, Zn: 1.65, and Cd: 1.69, respectively. The large electronegativity difference drives electron transfer from M to O atoms, resulting in significant ionic character in the M–O bonds. To directly examine the spatial features of charge redistribution, we calculate the electron localization function (ELF) and perform Bader charge analysis. The ELF quantifies the degree of electron localization, with values approaching 1 indicating stronger localization [21]. Figure 1d illustrates the evolution of electron localization from BeO to CdO monolayers using 2D ELF slices. For all MO monolayers, the ELF distributions suggest mixed ionic–covalent bonds, with high ELF regions present both in the M–O bonding region and around the O atoms. In the Group IIA monolayers, extremely high ELF values (red, ELF ≈ 1.0) are clearly evident in both regions. By contrast, in the Group IIB monolayers, the high ELF regions (yellow, ELF ≈ 0.8) are more strongly contracted around O atoms, while electron localization in the M–O bonding region is reduced. This redistribution indicates that, from Group IIA to Group IIB, the covalent contribution to the M–O bond decreases and the ionic character becomes more pronounced.

Quantitative Bader charge analysis indicates that the transferred charge from the metal to the O atom is 1.69, 1.63, 1.47, 1.18, and 1.19 e, corresponding to the series from BeO to CdO monolayers. The decrease in the Bader charge transfer does not directly follow the electronegativity difference trend (Figure 1c), suggesting that static charge transfer is influenced not only by M–O electronegativity difference but also by other factors, including bond length, atomic radius, the spatial distribution of charge density, and the underlying electronic structure. Notably, although the Group IIB metals (Zn and Cd) possess relatively higher electronegativity, their Bader charge transfer remains unexpectedly low (≈1.2 e) due to the strong shielding of the valence s electrons by the filled d10 shell.

Electronegativity differences, along with ELF and Bader charge analyses, demonstrate that all M–O bonds exhibit a mixed ionic–covalent character. The d^10^ configuration of the Group IIB elements strongly influences charge transfer and electron localization behavior. These static properties form the physical basis for interpreting the dielectric response. Nevertheless, the macroscopic dielectric response is inherently dynamic. It encompasses not only the static charge distribution but also the charge redistribution induced by atomic displacements.

### 3.2. Low-Frequency Dielectric Response: Phonon Dispersion and Born Effective Charge

In the low-frequency limit, the dielectric response of materials is dominated by ionic polarization. Understanding this response requires examining two quantities that are intimately connected with lattice dynamics. The phonon spectrum reveals the lattice vibrational modes and their frequencies as a function of the wave vector, thereby providing the dynamical basis of the polarization. The BECs quantify the polarization induced per unit atomic displacement. Before carrying out these analyses, the mechanical and dynamic stability of the materials should be assessed based on their elastic constants and phonon spectrum. For the MO monolayers, the in-plane elastic constant matrix can be written as

(1) $$\left[C\right]=\left(\begin{array}{ccc} C_{11} & C_{12} & 0\\ C_{12} & C_{22} & 0\\ 0 & 0 & C_{66} \end{array}\right)$$

where *C*_11_, *C*_22_ and *C*_66_ are the independent elastic constants of the 2D lattice. Table 1 lists the calculated elastic constants of the five MO monolayers, all of which meet *C*_11_ > 0, *C*_66_ > 0 and *C*_11_·*C*_22_ > *C*_12_·*C*_12_, fulfilling the Born–Huang criteria [22,23], confirming that all monolayers are mechanically stable.

**Figure 2:**
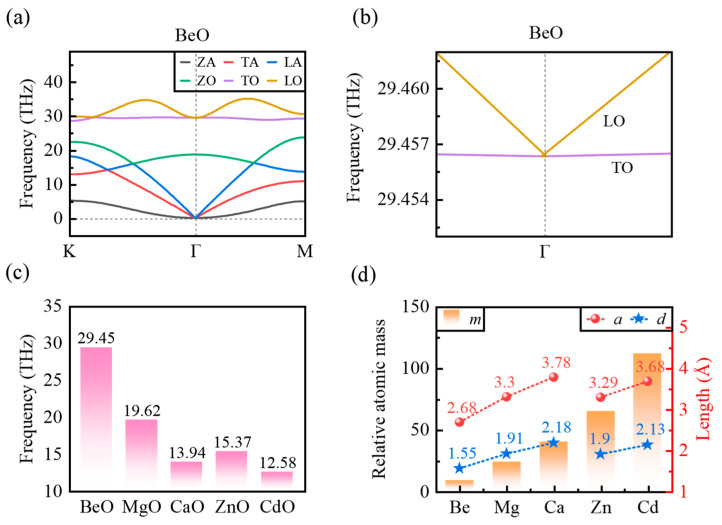
(**a**) Phonon dispersion of the BeO monolayer. The six curves correspond to the out-of-plane acoustic (ZA, gray), transverse acoustic (TA, red), longitudinal acoustic (LA, blue), out-of-plane optical (ZO, green), transverse optical (TO, purple) and longitudinal optical (LO, yellow) branches, respectively. (**b**) Enlarged views of LO and TO phonons in the BeO monolayer near the Γ point (from Figure (**a**)). (**c**) The TO phonon resonance frequency. (**d**) The relative atomic mass *m* of the metal atoms, the lattice constant *a* and the bond length *d* of the MO monolayers.

Dynamical stability describes a crystal structure’s resistance to small displacements from its atomic equilibrium positions. It is typically evaluated from the phonon spectrum. A structure is dynamically stable if no imaginary phonon modes (often plotted as negative frequencies) occur. As presented in Figure S1, the phonon dispersion curves of BeO, MgO, CaO, ZnO, and CdO monolayers exhibit no negative frequencies, confirming their dynamical stability. Furthermore, the long-wavelength optical phonons reveal a key feature relevant to the low-frequency dielectric response: all monolayers display the degeneracy of the longitudinal optical (LO) and transverse optical (TO) phonons at the Γ point (Figure 2a shows BeO as an example; see Figure S1 for the Supplementary Materials). This behavior distinguishes it from both 3D polar crystals and non-polar materials in any dimension.

In 3D polar materials (Figure S2a), the LO and TO phonons exhibit a pronounced LO–TO splitting at the Γ point. Physically, the ionic displacement associated with LO phonon generates a macroscopic polarization density, which in turn produces a macroscopic electric field. This field arises from long-range Coulomb (dipole–dipole) interactions and provides an additional restoring force for the LO phonon. Consequently, the LO phonon frequency increases near the Γ point, causing the LO–TO splitting [24]. In non-polar materials, by contrast, lattice symmetry ensures LO–TO degeneracy at the Γ point. The optical branches are analytic with zero slope (Figure S2b). Remarkably, the polar monolayers of BeO, MgO, CaO, ZnO, and CdO exhibit a third distinctive behavior for the LO and TO branches at and near the Γ point (Figure 2b shows BeO as an example; see Figure S3 for the Supplementary Materials). Although these monolayers are intrinsically polar, the 2D confinement prevents the macroscopic electric field that causes LO–TO splitting from being sustained at the Γ point, so that the LO and TO phonon modes become degenerate. Once the phonon wavevector deviates from the Γ point, the long-range Coulomb interaction manifests as a nonanalytic term, giving rise to a linear LO phonon dispersion with a finite group velocity and a characteristic V-shaped dispersion. The phenomenon has been observed in other 2D polar materials [7,25].

This unconventional LO–TO splitting is controlled by wavevector-dependent screening, with the key factor being the distinctive form of the effective 2D dielectric function [26,27]:

(2) $$\varepsilon_{2D}(|q_{p}|)=\varepsilon_{\mathrm{ext}}+r_{\mathrm{eff}}|q_{p}|$$

where $\varepsilon_{\mathrm{ext}}$ is the effective dielectric constant of the surrounding medium (for a free-standing monolayer in vacuum, $\varepsilon_{\mathrm{ext}}=1$). The parameter $r_{\mathrm{eff}}$ describes the material’s intrinsic screening capability, which depends on its dielectric properties and effective thickness. Due to dimensional effects, the relationship between the squared frequencies of the LO and TO phonons satisfy

(3) $$\omega_{\mathrm{LO}}^{2}\approx \omega_{\mathrm{TO}}^{2}+S|q_{p}|/\varepsilon_{2D}(|q_{p}|)$$

Here, *S* is a material-dependent constant related to the effective charges, atomic masses and other factors. In the long-wavelength limit, the momentum $q_{p}$ tends to zero, the dielectric function simplifies to $\varepsilon_{2D}(|q_{p}|)=\varepsilon_{\mathrm{ext}}=1$, which leads to $\omega_{\mathrm{LO}}^{2}\approx \omega_{\mathrm{TO}}^{2}$. Hence, degeneracy of the LO and TO phonons is found at the Γ point. When $q_{p}$ increases from zero but remains small ($q_{p}<<\varepsilon_{\mathrm{ext}}r_{\mathrm{eff}}^{-1}$, near the Γ point), $\varepsilon_{2D}(|q_{p}|)\approx \varepsilon_{\mathrm{ext}}$. The frequency expression can then be simplified to $\omega_{\mathrm{LO}}^{2}\approx \omega_{\mathrm{TO}}^{2}+(S/\varepsilon_{\mathrm{ext}})*|q_{p}|$, showing that the square of the LO phonon frequency has a linear relationship with the magnitude of momentum $\left|q_{p}\right|$ with a finite dispersion slope of $S/2\varepsilon_{\mathrm{ext}}\omega_{\mathrm{TO}}$. This unique behavior constitutes remarkable dielectric properties in 2D polar systems, distinguishing them from other material classes.

In addition, the TO phonon frequency at the Γ point also exhibits a clear trend with the metal atomic number. Specifically, as demonstrated in Figure 2c, TO phonon frequency decreases monotonically with increasing atomic number. For Group IIA monolayers, the frequency drops from 29.45 THz (BeO) to 13.94 THz (CaO), whereas for Group IIB monolayers, it decreases from 15.37 THz (ZnO) to 12.58 THz (CdO). The corresponding resonance wavelengths for BeO, MgO, CaO, ZnO, and CdO monolayers are 10.19 μm, 15.29 μm, 21.52 μm, 19.52 μm and 23.85 μm, respectively, all falling within the mid- to far-infrared spectral range. This phonon softening originates primarily from the combined effects of the increased vibrational inertia associated with heavier cations, weakened interatomic interactions due to lattice expansion and longer bond lengths (Figure 2d), and corresponding changes of the electronic structure [28,29]. From the perspective of dielectric response, the softening of the TO phonon lowers the characteristic frequency of the ionic polarization, thereby shifting the resonance peak in the dielectric function in the infrared to longer wavelengths.

The phonon analysis above identifies the lattice vibrational modes and frequencies in 2D polar materials, which are related to dielectric response. Nevertheless, a quantitative understanding of the microscopic origin of displacement-induced polarization and its contribution to the dielectric response requires an additional key physical quantity: BECs [30]. Under zero macroscopic electric field, the BECs establish a fundamental link between the lattice dynamics and dielectric properties of materials. Generally, a larger BEC signifies a stronger polarization response induced by ionic displacements. This enhanced polarization consequently increases the material’s overall dielectric response. Using DFPT, we compute the BECs for all atoms in the MO monolayers: the results are summarized in Table 2.

As shown in Table 2, the BECs of the MO monolayers satisfy the acoustic sum rule ($Z_{M}^{*}+Z_{O}^{*}=0$). However, their magnitudes deviate from the nominal ionic charges of ±2 e, which stems from the partial covalency of the M–O bonds. The BECs capture not only the nominal static ionic charge but also the dynamic redistribution of the electron cloud in response to ionic displacement. This redistribution stems from changes in orbital hybridization, which is visually captured in the projected density of states of the five monolayers (Figure S4). It directly illustrates the progressive evolution of d orbital contributions near the band edges. For the Group IIA monolayers (BeO and MgO), the unoccupied d-states of the cations lie far above the O-2p states, making p–d hybridization negligible. Hence, the covalent character in the two monolayers stems predominantly from s–p hybridization between the cation and oxygen. Upon ionic displacement, the incomplete dynamic redistribution of these bonding electrons partially cancels the core displacement dipole, reducing the BEC below 2 e (Be: 1.97 e; Mg: 1.84 e), corresponding to a weaker ionic polarization response. In contrast, the notably larger BEC of Ca (2.35 e) originates from the fact that the low-lying unoccupied 3d states actively participate in p–d hybridization at the band edge. This interaction provides an efficient channel for additional dynamic charge transfer upon ionic displacement, which generates an extra dipole moment and thereby strengthens ionic polarization. For the Group IIB monolayers, Zn and Cd possess filled d^10^ configurations (Zn-3d^10^, Cd-4d^10^). Their even larger BECs (Zn: 2.48 e; Cd: 3.05 e) are due to strong d–p hybridization between the occupied metal d orbitals and O-2p orbitals. This hybridization induces substantial electron-density rearrangement during ionic displacement, yielding large dynamical charges and the strongest ionic polarization response. The larger BEC of Cd compared with Zn is attributed to the shallower energy level of Cd-4d orbitals relative to the Zn-3d orbitals, which enhances orbital overlap and facilitates dynamical charge transfer.

Overall, d-orbital involvement increases progressively from negligible p–d hybridization in Be/Mg, to active participation of low-lying empty 3d states in Ca, and further to strong p–d hybridization from the filled d^10^ shells in Zn/Cd. This trend promotes a systematic increase in the BECs. The p–d hybridization mechanism for enhancing BECs has been reported in previous studies [31,32,33]. Combined with the softening of TO phonon at the Γ point (Figure 2c) and the variation of reduced mass and unit cell area, these factors collectively enhance the ionic dielectric response across the MO series. The quantitative decomposition of each factor’s contribution identifies phonon softening as the primary driver of the enhanced response, the Born effective charge as a significant secondary contribution, and the reduced mass and unit cell area as moderating influences (Tables S2 and S3). Moreover, the BECs exhibit pronounced anisotropy between the in-plane and out-of-plane directions. As listed in Table 2, the in-plane components ($Z_{xx}^{*}=Z_{yy}^{*}$) significantly exceed the out-of-plane component ($Z_{zz}^{*}$) for all monolayers (e.g., 3.05 vs. 0.40 for CdO). This anisotropy stems directly from the strong in-plane chemical bonds versus the weak out-of-plane coupling, indicating that the ionic contribution to the dielectric response in the infrared regime is dominated almost entirely by in-plane polarization. We next examine the dielectric response in the high-frequency (optical) regime. When the frequency of the external field far exceeds the characteristic lattice-vibration frequencies, ions cannot follow the rapid variation of the electric field due to their inertia. Consequently, the polarization response is dominated by electronic contributions and is described by the frequency-dependent complex dielectric function.

### 3.3. High-Frequency Dielectric Response: Complex Dielectric Function and Optical Property

**Figure 3:**
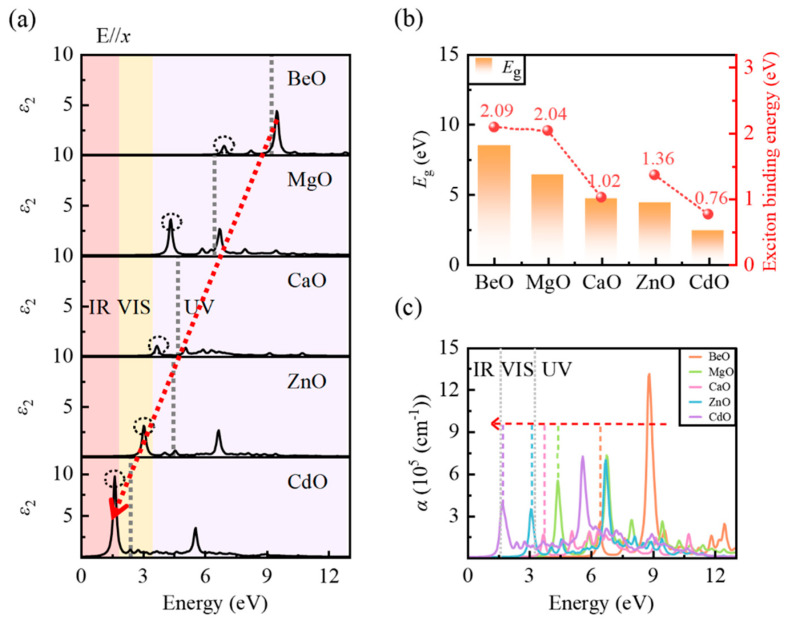
(**a**) The imaginary part of the complex dielectric function, (**b**) the quasiparticle band gap value derived from *GW*_0_ calculations (represented by orange bars) and the exciton binding energy, (**c**) the absorption coefficient for the MO monolayers. In Figure (**a**), pink, yellow and purple areas correspond to the infrared (IR), visible (VIS), and ultraviolet regions (UV), respectively. The red dashed line indicates that the overall imaginary part exhibits a redshift trend as the atomic number of the metal increases. The red dotted line in Figure (**c**) also indicates the redshift of absorption spectrum.

**Figure 4:**
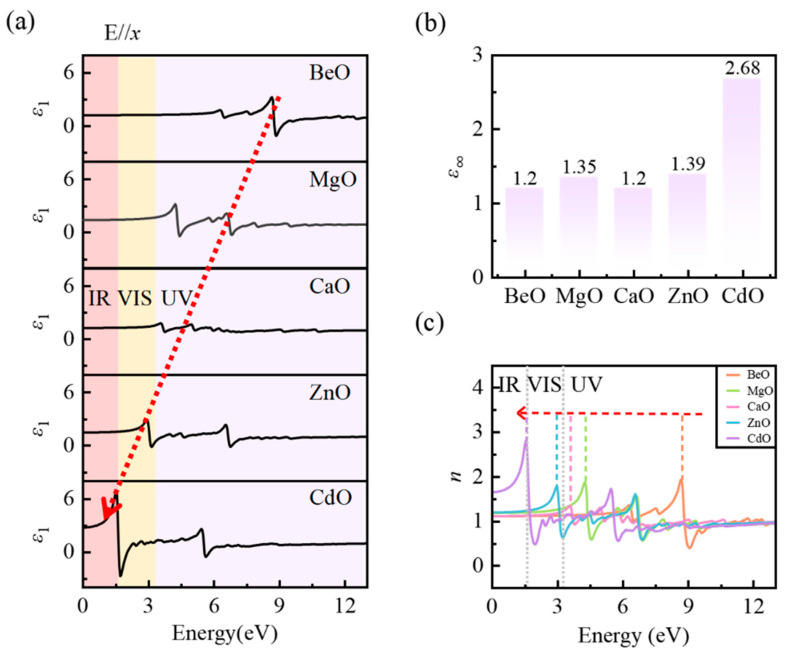
(**a**) The real part of the complex dielectric function $\varepsilon_{1}(\omega )$, (**b**) the optical dielectric constant, and (**c**) the refractive index for the MO monolayers.

We employ the *GW*_0_ + BSE approach to compute the complex dielectric function $\varepsilon (\omega )=\varepsilon_{1}(\omega )+i\varepsilon_{2}(\omega )$. The real part $\varepsilon_{1}(\omega )$ reflects the dispersive, energy-storing polarization response, whereas the imaginary part $\varepsilon_{2}(\omega )$ corresponds to the absorptive (dissipative) component. *ω* denotes the photon frequency. It is worth noting that the dielectric function via the *GW*_0_ + BSE approach captures only the electronic polarization, as BSE is solved for fixed nuclear positions. This is appropriate for the optical-frequency range, where the ionic contribution is negligible [34]. Furthermore, key optical properties such as the absorption coefficient and refractive index can be derived from ε(ω) through well-established analytical relationships, thus offering complementary insights into the frequency-dependent electronic dielectric response. Figure 3 and Figure 4 present the high-frequency dielectric response of the five MO monolayers dominated by electronic polarization and its evolution with increasing metal atomic number. Figure 3 highlights $\varepsilon_{2}(\omega )$ and the optical absorption coefficient α(ω), whereas Figure 4 details $\varepsilon_{1}(\omega )$ and the refractive index n(ω). As all monolayers are in-plane isotropic, only the case with E//*x* is shown.

Figure 3a presents the imaginary part $\varepsilon_{2}(\omega )$ for the MO monolayers. With increasing metal atomic number, the absorption onset and the prominent peaks in $\varepsilon_{2}(\omega )$ redshift from BeO to CdO, extending the optical response from the deep-ultraviolet to the near-infrared (the convergence with respect to the k-mesh is verified in Figure S5). This evolution closely follows the reduction of the *GW*_0_ quasiparticle band gap (Figure 3b), which decreases from 8.5 eV (BeO) to 2.4 eV (CdO). Notably, the dominant peak intensity exhibits completely opposite trends in group IIA and group IIB elements (Figure 3a and Table 3). For group IIA, the dominant peak intensity monotonically decreases with increasing atomic number. This trend is primarily attributed to the elongation of the M–O bond and the increased diffuseness of the metal valence orbitals along the series. The reduced spatial overlap between these metal orbitals and the O-2p states leads to a decrease in the optical transition matrix elements, consequently weakening the transition strength. In contrast, within group IIB, the dominant peak of CdO is stronger than that of ZnO. This is primarily because the stronger p–d repulsion in CdO reduces the band gap, so that the dominant transitions occur at lower energies and are amplified by the $1/\omega^{2}$ factor. In addition, pronounced bound-exciton peaks (indicated by black dashed circles) are observed for all monolayers in the vicinity of the corresponding quasiparticle gaps (gray dashed lines in Figure 3a). This feature originates from the explicit inclusion of the electron–hole Coulomb interaction, enabling a more accurate description of excitonic effects [35]. The exciton binding energy is

(4) $$E_{B}=E_{g}-E_{\mathrm{ex}}$$

where $E_{B}$ is the exciton binding energy, $E_{g}$ is the *GW*_0_ quasiparticle band gap and $E_{\mathrm{ex}}$ denotes the energy of the observed exciton peak. As shown in Figure 3b, the exciton binding energies of the MO monolayers are generally on the order of eV, far exceeding those of the corresponding bulk counterparts (typically only several tens of meV) [36,37]. This enhancement is mainly due to the reduction in dielectric shielding caused by quantum confinement, which in these monolayers is dominated by the in-plane electronic polarizability [38,39]. Furthermore, within each family (IIA or IIB), the exciton binding energy decreases with increasing metal atomic number (Figure 3b). This trend is because the narrowing band gap enhances the in-plane electronic polarizability and thus the dielectric screening, which weakens the electron–hole Coulomb interaction [40].

Figure 3c displays the absorption spectra of the MO monolayers. The absorption coefficient α(ω) is determined by both the real and imaginary parts. Consistent with the evolution of $\varepsilon_{2}(\omega )$, the absorption onset and major peaks redshift progressively from the deep-ultraviolet (BeO) to the near-infrared (CdO) as the metal atomic number increases. The obtained absorption coefficients reach the order of 10^5^–10^6^ cm^−1^, surpassing those of the corresponding bulk oxides (typically ∼10^4^ cm^−1^) by 1–2 orders of magnitude [41]. This significant enhancement is due to the intrinsic quantum confinement effect in the 2D structure. Notably, the peak distribution in $\varepsilon_{2}(\omega )$ (Figure 3a) differs markedly from that in α(ω) (Figure 3c). For all monolayers except BeO (MgO, CaO, ZnO, and CdO), the relative intensities of the characteristic peaks are reversed between the two spectra (Table 3 and Table 4). Taking MgO as an example, the dominant peak in $\varepsilon_{2}(\omega )$ at 4.36 eV corresponds to a low-energy excitonic resonance. In contrast, in the absorption spectrum α(ω), a higher-energy secondary transition at 6.77 eV becomes the main absorption peak and exhibits a larger absorption coefficient. This inversion indicates that optical absorption and dielectric response are not related through a simple one-to-one, monotonic correspondence. Specifically, the quantum confinement effect strengthens the electron–hole Coulomb interaction, giving rise to pronounced excitonic effects that dominate $\varepsilon_{2}(\omega )$ at low energies. In contrast, for $\alpha (\omega )=\varepsilon_{2}(\omega )\cdot \omega /\left[n(\omega )c\right]$, the explicit *ω* prefactor amplifies higher-energy transitions, while the frequency-dependent refractive index n(ω) provides additional modulation. These two effects together cause the higher-energy continuum-state peaks to surpass the exciton resonance in absorption intensity. BeO is an exception. Its ultra-wide gap means the $\varepsilon_{2}$ peak at 8.77 eV already arises from continuum transitions, so no intensity inversion occurs upon conversion to α(ω).

As discussed above regarding $\varepsilon_{2}(\omega )$, the resonant structures (absorption peaks) arise from different electronic transition processes. According to the Kramers–Kronig relations, these resonances in $\varepsilon_{2}(\omega )$ determine the dispersive behavior of $\varepsilon_{1}(\omega )$. $\varepsilon_{1}(\omega )$ and $\varepsilon_{2}(\omega )$ collectively characterize the optical-frequency dielectric response dominated by electronic polarization. Figure 4a presents the real part $\varepsilon_{1}(\omega )$ of the MO monolayers. The peaks in $\varepsilon_{1}(\omega )$ appear near the frequencies of specific electronic transitions, where the in-phase electronic polarization response is significantly enhanced. Similar to the case for $\varepsilon_{2}(\omega )$, as the metal atomic number increases and the band gap decreases, the features in $\varepsilon_{1}(\omega )$ also exhibit a redshift from the deep-ultraviolet to the near-infrared. From Table 5, the dominant peak intensity in $\varepsilon_{1}(\omega )$ exhibits opposite trends for the IIA and IIB series, which agrees with the trend reported in $\varepsilon_{2}(\omega )$ (Table 3). This agreement stems from the Kramers–Kronig relations, linking the amplitude of a dispersive peak in $\varepsilon_{1}(\omega )$ directly to the integrated strength and spectral position of its corresponding absorption feature in $\varepsilon_{2}(\omega )$. Notably, the energies of both the main and second peaks in $\varepsilon_{1}(\omega )$ are lower than those of the corresponding peaks in $\varepsilon_{2}(\omega )$ by approximately 0.08–0.12 eV (Table 3 and Table 5). For example, in MgO the main peaks of $\varepsilon_{1}(\omega )$ and $\varepsilon_{2}(\omega )$ occur at 4.24 and 4.36 eV, respectively, while the second peaks occur at 6.61 and 6.73 eV. This ~0.1 eV shift is a universal feature of the dielectric response near resonances, which is dictated by the Kramers–Kronig relations.

Figure 4b presents the electronic (high-frequency) dielectric constant $\varepsilon_{\infty }$ of the MO monolayers. $\varepsilon_{\infty }$ quantifies the purely electronic contribution to the dielectric response at frequencies above the phonon range. Because the *GW*_0_ + BSE approach does not account for lattice dynamics, the calculated $\varepsilon_{\infty }$ contains only electronic polarization and excludes ionic polarization. The $\varepsilon_{\infty }$ values of BeO, MgO, CaO, and ZnO are confined to a narrow range of 1.2–1.4. This reflects the weak electronic dielectric response typical of wide-band-gap monolayers. In contrast, CdO exhibits a much larger $\varepsilon_{\infty }$ of 2.68 (about twice that of the others), indicating a substantially enhanced electronic dielectric response. This follows from the Kramers–Kronig integral, in which a 1/*ω*′ kernel strongly weights low-energy excitations, making $\varepsilon_{\infty }$ sensitive to both the energy position and spectral weight of the peaks. For BeO, MgO, CaO, and ZnO, the increase in low-energy weighting induced by the redshift of the main peak is largely compensated by a concurrent reduction in transition strength, keeping $\varepsilon_{\infty }$ within the narrow range of 1.2–1.4. CdO differs in that its lowest-energy excitonic resonance combines both a large 1/*ω*′ weight and a strong peak intensity, and their combined effect markedly enhances $\varepsilon_{\infty }$.

This redshift, spanning from the deep-ultraviolet in BeO to the near-infrared in CdO, is also reflected in the refractive index n(ω) (Figure 4c). As n(ω) is governed by the complex dielectric function, its spectral evolution directly reveals the frequency-dependent electronic dielectric response. The relationship between the refractive index and the dielectric function is

(5) $$n(\omega )=\sqrt{\frac{{\left(\varepsilon_{1}^{2}+\varepsilon_{2}^{2}\right)}^{1/2}+\varepsilon_{1}}{2}}$$

In the zero-frequency limit, $\varepsilon_{2}(\omega )$ approaches zero, and Equation (5) reduces to $n(0)=\sqrt{\varepsilon_{1}(0)}=\sqrt{\varepsilon_{\infty }}$. As summarized in Table 6, the static refractive indices of five MO monolayers are 1.09 (BeO), 1.16 (MgO), 1.09 (CaO), 1.18 (ZnO), and 1.64 (CdO), which are consistent with the $\varepsilon_{\infty }$ values in Figure 4b through the relation $n(0)=\sqrt{\varepsilon_{\infty }}$. The static refractive index of CdO is markedly higher than those of the other materials, further confirming its strongly enhanced electronic polarization response. It is worth noting that in our *GW*_0_ + BSE calculations, the spin–orbit coupling effect is not included. Since the CBM derives from metal s orbitals in all five MO monolayers, the spin–orbit coupling effect affects only the valence band side. The expected corrections are confined to the order of tens of meV. This is negligibly small relative to the several-eV band gap variation across the series and insufficient to alter any of the trends we have discussed above. In addition, good agreement with literature data [9,10,11,12,42] for several representative properties, including band gaps, $\varepsilon_{\infty }$ and TO phonon frequencies (Table S1), combined with the structural comparison presented earlier, provides strong support for the overall reliability of our findings. Furthermore, although all optical properties are obtained directly from the 3D supercell output, the vacuum-independent 2D quantities confirm that all comparative trends are correct (Figure S6).

In summary, the present study systematically elucidates the dielectric properties of the MO monolayers and establishes a complete dielectric spectrum spanning low-frequency ionic polarization to high-frequency electronic polarization, which provides an essential theoretical foundation and direct guidance for their potential applications. These monolayers adopt the same crystal structure as h-BN and are therefore well suited for the fabrication of sliding ferroelectric devices. The distinct dielectric responses of CdO monolayers, including strong ionic and electronic dielectric responses among the series, are advantageous for serving as high-*κ* gate dielectrics in field-effect transistors and enhancing light confinement in nanophotonic devices. In contrast, the weak dielectric screening exhibited by wide-bandgap BeO and MgO monolayers is beneficial for enhancing the tunneling electroresistance in ferroelectric tunnel junctions. In addition, the broad coverage of absorption edges from the deep-ultraviolet (BeO) to the near-infrared (CdO) makes these materials promising candidates for broadband photodetection.

## 4. Conclusions

We studied the broadband dielectric response of five MO monolayers (BeO, MgO, CaO, ZnO, and CdO) from ionic to electronic polarization using DFPT and *GW*_0_ + BSE calculations. In the low-frequency regime, all monolayers exhibit a typical 2D polar feature that LO–TO degeneracy at the Γ point accompanied by a V-shaped nonanalytic LO phonon dispersion. With increasing metal atomic number, phonon softening is observed. Simultaneously, the Born effective charges increase from 1.97 e (Be) to 3.05 e (Cd). This trend originates from the evolving d-orbital hybridization: negligible p–d interaction in Be/Mg, significant participation of low-lying unoccupied 3d states in Ca, and strong p–d hybridization from filled d^10^ configurations in Zn and Cd. This cooperative interplay between phonon softening, reduced mass, and unit cell area, coupled with enhanced dynamical charge transfer, strengthens the ionic contribution across the series. In the high-frequency regime, the *GW*_0_ quasiparticle band gap decreases from 8.5 eV (BeO) to 2.4 eV (CdO), driving a pronounced redshift of the optical dielectric function from the deep-ultraviolet to the near-infrared. CdO exhibits the strongest electronic polarization among five monolayers, manifested in its highest optical dielectric constant (2.68, roughly twice that of others) and largest static refractive index (1.64), arising from the combined large spectral weight and high intensity of its lowest-energy excitonic resonance. Interestingly, the dominant peak intensity in $\varepsilon_{1}(\omega )$ and $\varepsilon_{2}(\omega )$ exhibits opposite trends between the two groups: it decreases monotonically across Group IIA due to reduced M–O orbital overlap, whereas within Group IIB, CdO surpasses ZnO owing to stronger p–d repulsion. These results establish a complete broadband dielectric spectrum for 2D MO monolayers, offering theoretical guidance for polarization engineering and the rational design of 2D ferroelectric devices.

## Figures and Tables

**Table 1:** Elastic constants of the MO monolayers (N/m).

	*C*_11_	*C*_22_	*C*_12_	*C*_66_	*C*_11_ × *C*_22_	*C*_12_ × *C*_12_
BeO	148	148	56	46	21,904	3136
MgO	82	82	50	16	6724	2500
CaO	57	57	40	8	3249	1600
ZnO	88	88	60	14	7744	3600
CdO	64	64	50	7	4096	2500

**Table 2:** Born effective charges (BECs) $Z^{*}$ of the M and O atoms (in units of e).

Be	Mg	Ca	Zn	Cd
$\left(\begin{array}{ccc} 1.97 & 0 & 0\\ 0 & 1.97 & 0\\ 0 & 0 & 0.50 \end{array}\right)$	$\left(\begin{array}{ccc} 1.84 & 0 & 0\\ 0 & 1.81 & 0\\ 0 & 0 & 0.62 \end{array}\right)$	$\left(\begin{array}{ccc} 2.35 & 0 & 0\\ 0 & 2.35 & 0\\ 0 & 0 & 0.76 \end{array}\right)$	$\left(\begin{array}{ccc} 2.48 & 0 & 0\\ 0 & 2.48 & 0\\ 0 & 0 & 0.39 \end{array}\right)$	$\left(\begin{array}{ccc} 3.05 & 0 & 0\\ 0 & 3.05 & 0\\ 0 & 0 & 0.40 \end{array}\right)$
O	O	O	O	O
$\left(\begin{array}{ccc} -1.97 & 0 & 0\\ 0 & -1.97 & 0\\ 0 & 0 & -0.50 \end{array}\right)$	$\left(\begin{array}{ccc} -1.84 & 0 & 0\\ 0 & -1.81 & 0\\ 0 & 0 & -0.62 \end{array}\right)$	$\left(\begin{array}{ccc} -2.35 & 0 & 0\\ 0 & -2.35 & 0\\ 0 & 0 & -0.76 \end{array}\right)$	$\left(\begin{array}{ccc} -2.48 & 0 & 0\\ 0 & -2.48 & 0\\ 0 & 0 & -0.39 \end{array}\right)$	$\left(\begin{array}{ccc} -3.05 & 0 & 0\\ 0 & -3.05 & 0\\ 0 & 0 & -0.40 \end{array}\right)$

**Table 3:** Calculated quasiparticle band gaps and characteristic peak parameters of the imaginary part $\varepsilon_{2}$ for the MO monolayers.

Material	*GW*_0_ Band Gap (eV)	Main Peak Position (eV)	Main Peak Intensity	Second Peak Position (eV)	Second Peak Intensity
BeO	8.5	8.77	4.38	6.41	0.9
MgO	6.4	4.36	3.59	6.73	2.63
CaO	4.7	3.68	1.02	5.09	0.81
ZnO	4.4	3.04	3.06	6.69	2.59
CdO	2.4	1.64	9.61	5.57	3.47

**Table 4:** Characteristic peak parameters of the optical absorption coefficient (α) for the MO monolayers.

Material	Main Peak Position (eV)	α at Main Peak (10^5^ cm^−1^)	Second Peak Position (eV)	α at Second Peak (10^5^ cm^−1^)
BeO	8.85	13.11	6.45	2.42
MgO	6.77	7.29	4.40	5.48
CaO	5.09	1.86	3.68	1.60
ZnO	6.73	6.97	3.08	3.43
CdO	5.61	7.20	1.68	3.78

**Table 5:** Calculated quasiparticle band gaps and characteristic peak parameters of the real part $\varepsilon_{1}$ for the MO monolayers.

Material	*GW*_0_ Band Gap (eV)	Main Peak Position (eV)	Main Peak Intensity	Second Peak Position (eV)	Second Peak Intensity
BeO	8.5	8.69	3.23	6.32	1.77
MgO	6.4	4.24	3.15	6.61	2.09
CaO	4.7	3.56	1.73	4.97	1.61
ZnO	4.4	2.96	2.86	6.57	2.28
CdO	2.4	1.52	6.68	5.45	2.50

**Table 6:** Characteristic peak parameters of the refractive index (*n*) for the MO monolayers.

Material	*n*(0)	Main Peak Position (eV)	*n* at Main Peak	Second Peak Position (eV)	*n* at Second Peak
BeO	1.09	8.73	1.92	6.33	1.35
MgO	1.16	4.28	1.86	6.65	1.54
CaO	1.09	3.60	1.34	5.01	1.28
ZnO	1.18	3.00	1.76	6.61	1.59
CdO	1.64	1.56	2.79	5.49	1.71

## Data Availability

The data that support the findings of this study are available from the corresponding author upon reasonable request.

## Supplementary Materials

The following supporting information can be downloaded at: https://www.mdpi.com/article/10.3390/mi17050564/s1, Table S1. Comparison of selected calculated properties with previously reported values; Table S2. Calculated physical parameters in the MO monolayers, including Born effective charge, TO phonon frequency, lattice constant *a*, unit cell area *A*, reduced mass *μ*, ionic dielectric constant, and the predicted and DFPT ratios relative to BeO; Table S3. Decomposition of the ionic dielectric constant ratio into individual factor contributions relative to BeO for the MO monolayer series. Figure S1: The phonon dispersions of five MO monolayers, (a) BeO, (b) MgO, (c) CaO, (d) ZnO, (e) CdO; Figure S2: Comparative study of the LO and TO phonons in (a) 3D polar materials, (b) 3D/2D non-polar materials; Figure S3: Enlarged views of LO and TO phonons in five MO monolayer near the Γ point (from Figure S1), (a) BeO, (b) MgO, (c) CaO, (d) ZnO, (e) CdO; Figure S4: The projected density of states for the five MO monolayers, (a) BeO, (b) MgO, (c) CaO, (d) ZnO, (e) CdO; Figure S5: The imaginary part $\varepsilon_{2}(\omega )$ of the complex dielectric functions for the five MO monolayers with different k-meshes (7 × 7 × 1, 12 × 7 × 1 and 15 × 9 × 1). (a) BeO, (b) MgO, (c) CaO, (d) ZnO, (e) CdO. (f) Comparison of $\varepsilon_{2}(\omega )$ for all five MO monolayers at the 12 × 7 × 1 k-mesh. The infrared (IR), visible (VIS) and ultraviolet (UV) spectral regions are indicated by gray dashed lines; Figure S6: (a) The real part Re[α2D(ω)] and (b) the imaginary part Im[α2D(ω)] of the 2D polarizability, and the optical absorbance A(ω) for the five MO monolayers.

## Abbreviations

The following abbreviations are used in this manuscript:

2D	Two-dimensional
3D	Three-dimensional
BECs	Born effective charges
PAW	Projector Augmented Wave
VASP	Vienna Ab initio Simulation Package
DFPT	Density Functional Perturbation Theory
BSE	Bethe–Salpeter equation
ELF	Electron localization function
LO	Longitudinal Optical phonon
TO	Transverse Optical phonon
