# Supplementary material for: Broadband Dielectric Response of Group-II Metal Oxide Monolayers: From Ionic to Electronic Polarization
Source: Micromachines (Basel). 2026 May 1;17(5):564. doi: 10.3390/mi17050564 (PMC13208737; doi:10.3390/mi17050564)
Supplement: Supplementary file 1 [file micromachines-17-00564-s001.zip › micromachines-4240779-Supplementary.pdf]

Broadband Dielectric Response of Group-II Metal Oxide Monolayers: From Ionic to Electronic Polarization

*Pei Yin, Dongliang Jia, Dan Tan,* and Rusen Yang**

Table S1. Comparison of selected calculated properties with previously reported values

| Materials | $a_0$ (Å) | Band gap (eV) | $\varepsilon_\infty$ | TO frequency (THz) |
|---|---|---|---|---|
| BeO | 2.68 | 8.50($GW_0$) | 1.20 | 29.45 |
| | 2.673[a] | 6.70(HSE06)[a] | 1.162[a] | - |
| MgO | 3.30 | 6.40($GW_0$) | 1.35 | 19.62 |
| | 3.29[b] | 4.69(HSE06)[b] | - | - |
| CaO | 3.78 | 4.70($GW_0$) | 1.20 | 13.94 |
| | 3.783[c] | 2.619(PBE)[c] | 1.569[c] | - |
| ZnO | 3.29 | 4.40($GW_0$) | 1.39 | 15.37 |
| | 3.29[d] | - | 1.49[f] | 17.89[g] |
| CdO | 3.68 | 2.40($GW_0$) | 2.68 | 12.58 |
| | 3.689[e] | - | - | - |

[a]Ref. [1], [b]Ref. [2], [c]Ref. [3], [d]Ref [4], [e]Ref [5], [f]Ref. [6], [g]Ref. [7]

As shown in Table S1, the lattice constants of all five monolayers are in good agreement with previously reported values, and the deviations are within 1%. The band gaps obtained from our $GW_0$ calculations are larger than those from HSE06 or PBE, consistent with the established predictive hierarchy ($GW_0$ > HSE06 > PBE), as the $GW_0$ method captures many-body effects that are not accessible to standard density functionals. For instance, the $GW_0$ band gap of BeO (8.50 eV) exceeds the HSE06 value (6.70 eV) by ~27%, and the $GW_0$ band gap of CaO (4.70 eV) significantly exceeds the PBE value (2.619 eV). Importantly, both $GW_0$ (8.50 → 6.40 eV) and HSE06 (6.70 → 4.69 eV) consistently give a decreasing band gap from BeO to MgO. This preservation of the physical trend across theory levels, despite a systematic offset in absolute values, strongly supports the reliability of our $GW_0$ calculations. The calculated dielectric constants $\varepsilon_\infty$ and TO phonon frequencies are in reasonable overall agreement with literature values, with the observed discrepancies largely attributable to the different theories used (e.g., PBE tends

to overestimate the dielectric constant due to its band gap underestimation). Together, these results validate the reliability of our computational approach.

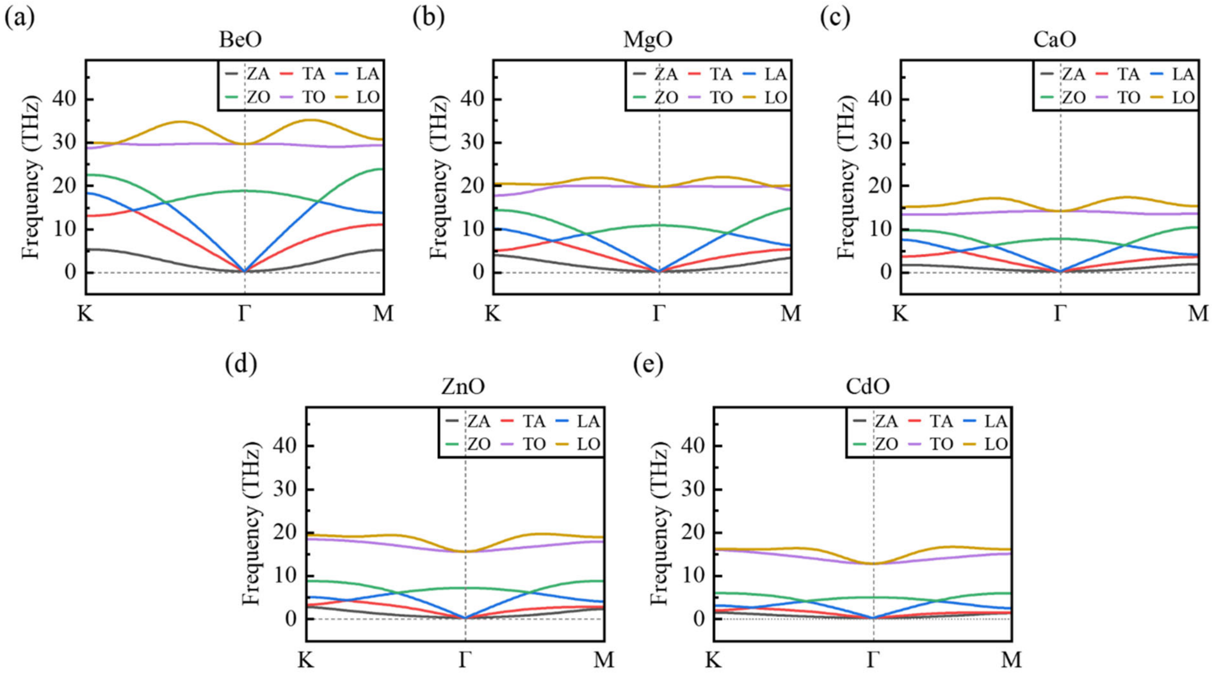


Figure S1. The phonon dispersions of five MO monolayers, (a) BeO, (b) MgO, (c) CaO, (d) ZnO, (e) CdO. The six curves correspond to the out-of-plane acoustic (ZA, gray), transverse acoustic (TA, red), longitudinal acoustic (LA, blue), out-of-plane optical (ZO, green), transverse optical (TO, purple) and longitudinal optical (LO, yellow) branches, respectively.

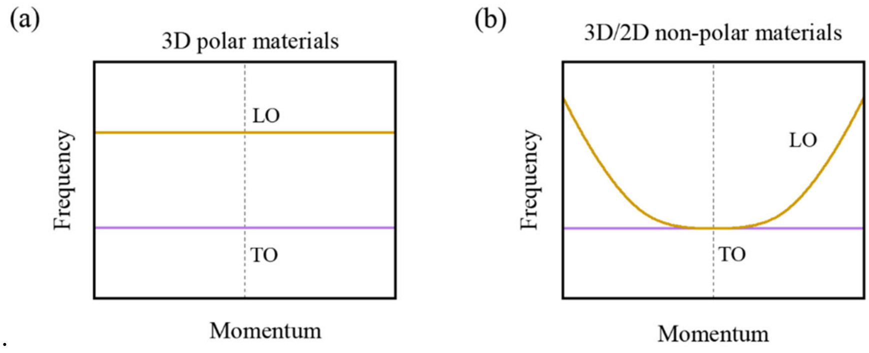


Figure S2. Comparative study of the LO and TO phonons in (a) 3D polar materials, (b) 3D/2D non-polar materials.

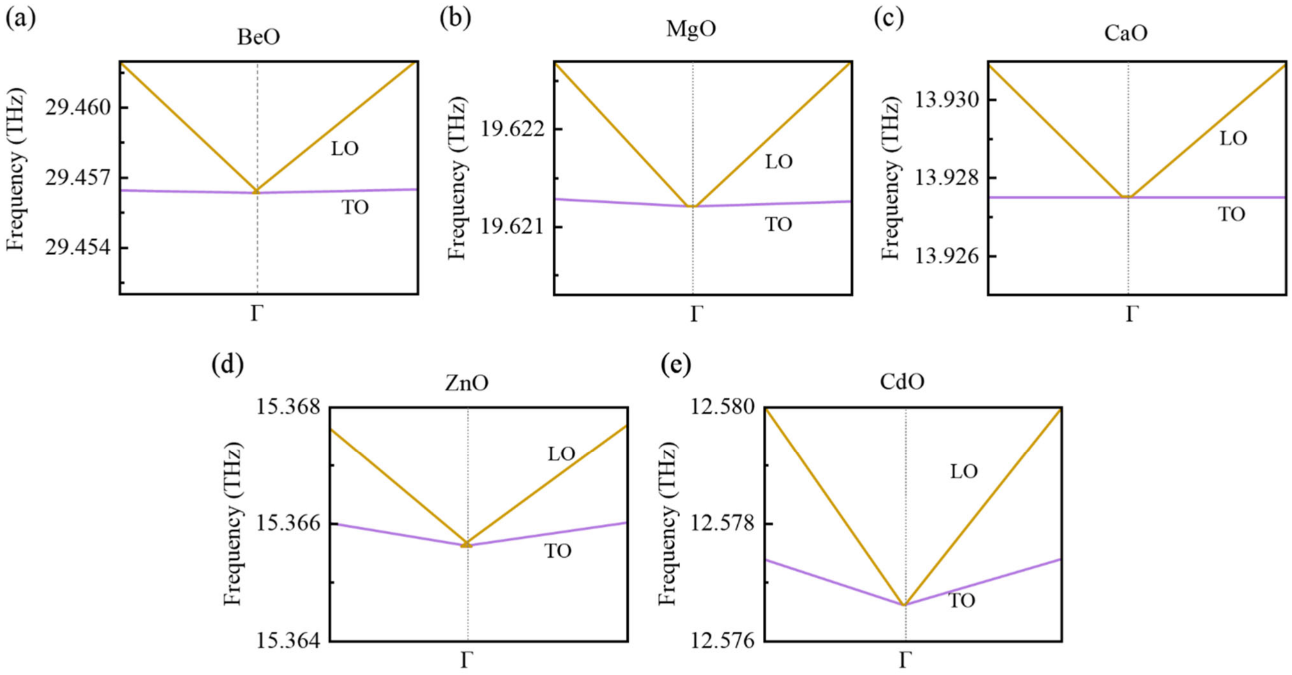


Figure S3. Enlarged views of LO and TO phonons in five MO monolayer near the Γ point (from Figure S1), (a) BeO, (b) MgO, (c) CaO, (d) ZnO, (e) CdO. These figures cover a small wave-vector range of $|\mathbf{q}_p| < 0.01$ Å$^{-1}$ from the Γ point along the K→Γ (left) and Γ→M (right) directions, respectively.

We calculate the projected density of states for the five MO monolayers and analyze the evolution of the d orbital contributions near the Fermi level and at the band edges. For BeO and MgO monolayers, the valence band maximum (VBM) is dominated by O-2p orbitals, showing negligible contribution from metal d orbitals. This observation aligns with weak p–d hybridization and relatively low Born effective charges of 1.97 e and 1.84 e. For CaO monolayer, unoccupied Ca-3d states appear near the conduction band minimum (CBM), providing a channel for hybridization with O-2p states and resulting in an increased Born effective charge of 2.35 e. For ZnO and CdO monolayers, the occupied Zn-3d and Cd-4d states lie near the VBM and exhibit significant overlap with O-2p states, which is responsible for the larger Born effective charges (2.48 e and 3.05 e, respectively). Therefore, the distribution of d states at the band edges and the strength of their hybridization with O-2p states are key factors governing the Born effective charge.

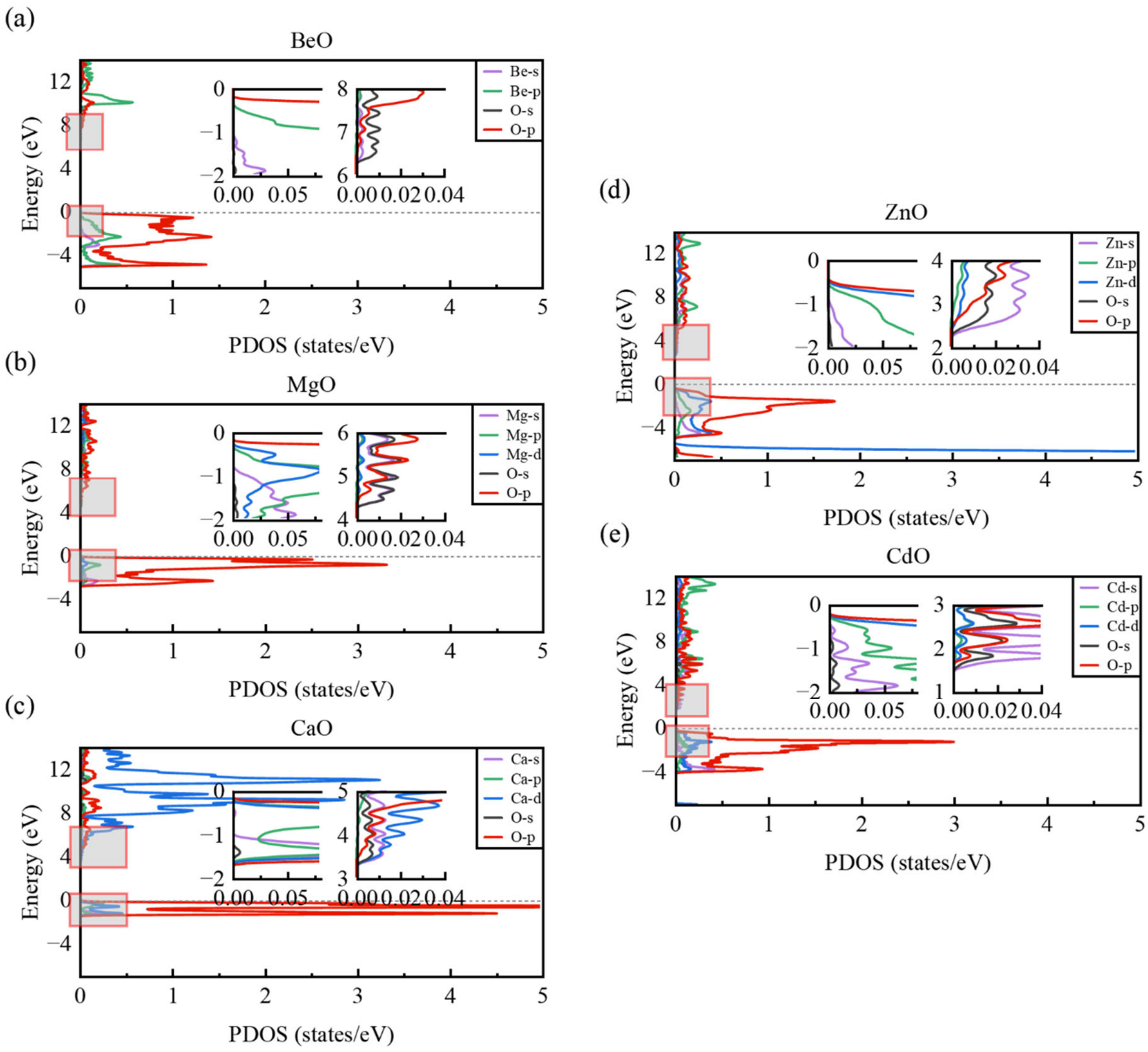


Figure S4. The projected density of states for the five MO monolayers, (a) BeO, (b) MgO, (c) CaO, (d) ZnO, (e) CdO. The Fermi level is set to 0 eV (indicated by the gray dashed line). In each subgraph, the upper and lower red rectangular boxes mark the conduction band minimum (CBM) and valence band maximum (VBM), respectively. The left inset shows a zoomed-in view of the VBM, while the right inset shows a zoomed-in view of the CBM.

Table S2. Calculated physical parameters in the MO monolayers, including Born effective charge $Z^*$, TO phonon frequency $\omega_{TO}$, lattice constant *a*, unit cell area *A*, reduced mass *μ*, ionic dielectric constant $\varepsilon_{\text{ion}}^{\text{DFPT}}$, and the predicted $R_{\text{pred}}$ and DFPT $R_{\text{DFPT}}$ ratios relative to BeO.

| Materials | $Z^*$ | $\omega_{TO}$ (THz) | $A$ (Å$^2$) | $\mu$ (u) | $\varepsilon_{\text{ion}}^{\text{DFPT}}$ | $R_{\text{pred}}$ | $R_{\text{DFPT}}$ |
|---|---|---|---|---|---|---|---|
| BeO | 1.97 | 29.45 | 6.22 | 5.77 | 0.27 | 1.00 | 1.00 |
| MgO | 1.84 | 19.62 | 9.43 | 9.65 | 0.22 | 0.78 | 0.81 |
| CaO | 2.35 | 13.94 | 12.37 | 11.44 | 0.44 | 1.61 | 1.63 |
| ZnO | 2.48 | 15.37 | 9.37 | 12.85 | 0.48 | 1.73 | 1.77 |
| CdO | 3.05 | 12.58 | 11.73 | 14.01 | 0.79 | 2.87 | 2.92 |

Table S3. Decomposition of the ionic dielectric constant ratio $R_{\text{pred}}$ into individual factor contributions relative to BeO for the MO monolayer series.

| Materials | $R_{Z^*}=\frac{(Z_X^*)^2}{(Z_{\text{BeO}}^*)^2}$ | $R_\mu=\frac{\mu_{\text{Beo}}}{\mu_X}$ | $R_A=\frac{A_{\text{Beo}}}{A_X}$ | $R_\omega=\frac{\omega_{\text{BeO}}^2}{\omega_X^2}$ | $R_{\text{pred}}$ |
|---|---|---|---|---|---|
| BeO | 1.00 | 1.00 | 1.00 | 1.00 | 1.00 |
| MgO | 0.87 | 0.60 | 0.66 | 2.25 | 0.78 |
| CaO | 1.42 | 0.50 | 0.50 | 4.46 | 1.61 |
| ZnO | 1.59 | 0.45 | 0.66 | 3.67 | 1.73 |
| CdO | 2.40 | 0.41 | 0.53 | 5.48 | 2.87 |

To quantitatively decompose the individual contributions of different factors to the ionic dielectric response, we introduce the following expression：

$$\Delta\varepsilon_{\text{ion}} \propto \frac{(Z^*)^2}{\mu \cdot A \cdot \omega_{\text{TO}}^2} \tag{1}$$

Here, $\Delta\varepsilon_{\text{ion}}$ denotes the enhancement of the ionic dielectric response， $Z^*$ is the Born effective charge, $\mu$ is the reduced mass of the M–O pair, *A* is the unit cell area, and $\omega_{\text{TO}}$ is the TO phonon frequency at the Γ point. This expression explicitly decomposes the variation in $\Delta\varepsilon_{\text{ion}}$ into four physically independent factors. As listed in Table S2, we first calculate these four parameters for the five MO monolayers and then use Equation (1) to estimate the

ionic dielectric constant ratio of each material relative to BeO, denoted as $R_{\text{pred}}$. At the same time, we directly calculate the corresponding ratio $R_{\text{DFPT}}$ by using DFPT. The close agreement between the two confirms the correctness of this decomposition scheme. To further clarify the relative contributions of the individual factors, we decomposed $R_{\text{pred}}$ into four independent factor ratios in Table S3. The results show that, from BeO to CdO, phonon softening is the dominant factor driving the enhancement of the ionic dielectric response (e.g., 5.48 for CdO). The increase in the Born effective charge provides a significant but secondary contribution (e.g., 2.40 for CdO). In contrast, the reduced mass and unit cell area have suppressive effects (e.g., 0.41 and 0.53 for CdO), partially offsetting the enhancement induced by the former two factors. These results indicate that the overall enhancement of the ionic dielectric response arises from the competition and collaboration among the four factors.

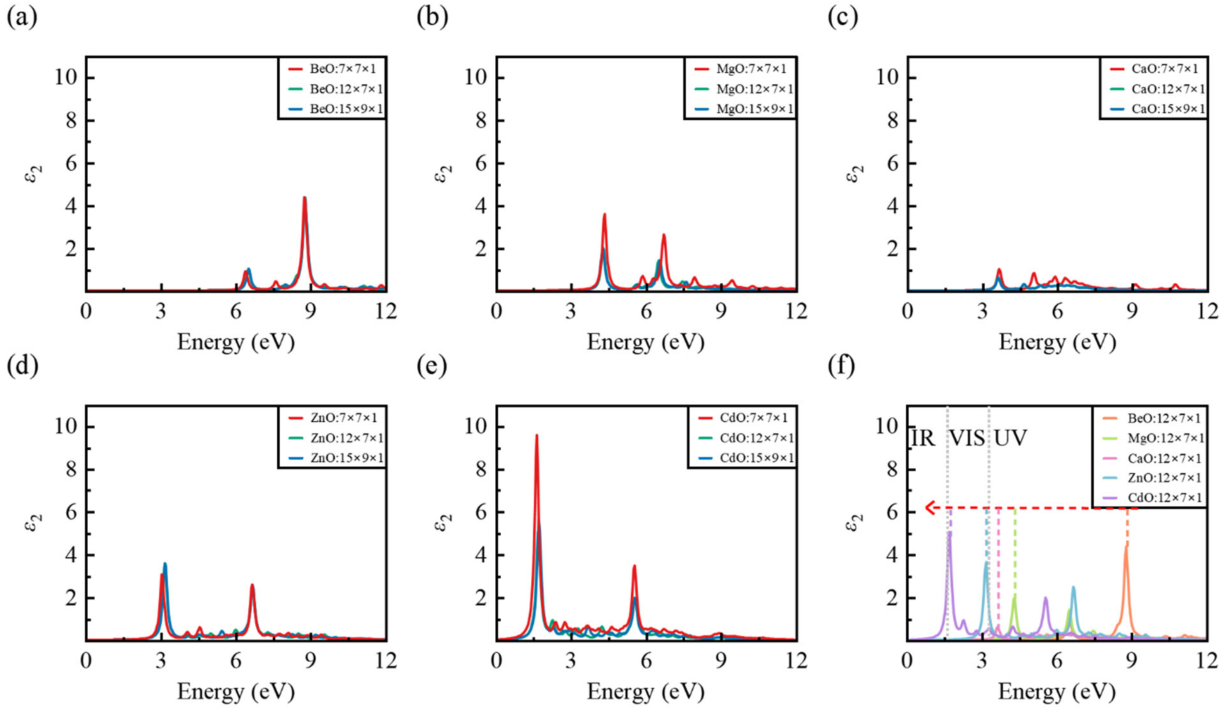


Figure S5. The imaginary part $\varepsilon_2(\omega)$ of the complex dielectric functions for the five MO monolayers with different k-meshes (7×7×1, 12×7×1 and 15×9×1). (a) BeO, (b) MgO, (c) CaO, (d) ZnO, (e) CdO. (f) Comparison of $\varepsilon_2(\omega)$ for five MO monolayers at the 12×7×1 k-mesh. The infrared (IR), visible (VIS) and ultraviolet (UV) spectral regions are indicated by gray dashed lines.

To examine the convergence of exciton binding energies and optical spectra, we present the imaginary part of the complex dielectric function for the five MO monolayers using different k-point meshes. As demonstrated in Figures S5(a-e), the calculated spectra for each material are nearly identical in peak position, although the peak intensities vary to some extent with increasing k-point density. Considering that the exciton binding energy is the

difference between the quasiparticle band gap and the exciton peak position, the well-converged peak positions at the 7×7×1 k-mesh indicate reliable binding energies. Figure S5f compares the $\varepsilon_2(\omega)$ of five MO monolayers at the 12×7×1 k-mesh, showing the same systematic redshift from BeO to CdO as obtained at the 7×7×1 k-mesh (Figure 3a and Table 3). This confirms the convergence of the 7×7×1 k-mesh for accurately capturing the key optical properties and overall physical trends in these monolayers.

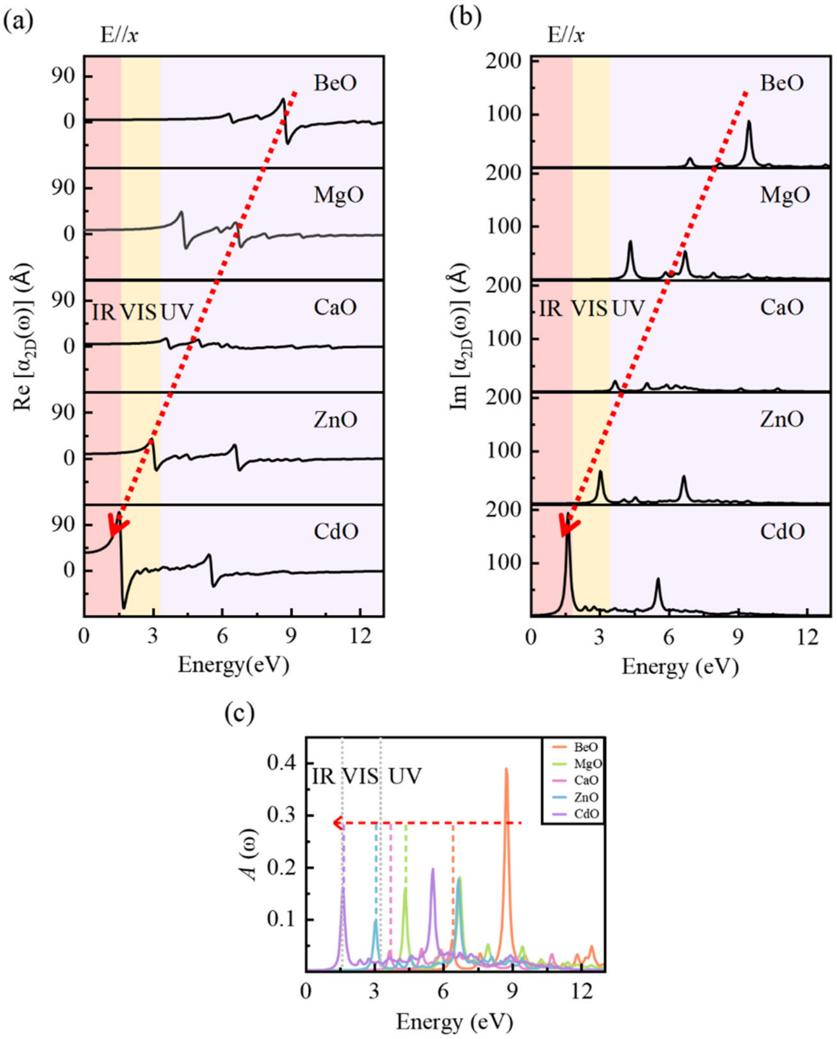


Figure S6. (a) The real part $\mathrm{Re}[\alpha_{2D}(\omega)]$ and (b) the imaginary part $\mathrm{Im}[\alpha_{2D}(\omega)]$ of the 2D polarizability, and the optical absorbance $A(\omega)$ for the five MO monolayers.

To further confirm our findings, we also calculate the vacuum-independent 2D polarizability $\alpha_{2D}(\omega)$ (both real part $\mathrm{Re}[\alpha_{2D}(\omega)]$ and imaginary part $\mathrm{Im}[\alpha_{2D}(\omega)]$), and the optical absorbance $A(\omega)$. These quantities are intrinsic to the 2D material. They are defined as:

$$\mathrm{Re}[\alpha_{2D}(\omega)] = [\varepsilon_1(\omega) - 1] \times L_z \qquad (2)$$

$$\text{Im}[\alpha_{2\text{D}}(\omega)] = \varepsilon_2(\omega) \times L_z \qquad (3)$$

$$A(\omega) = \frac{\omega}{c}\text{Im}[\alpha_{2\text{D}}(\omega)] \qquad (4)$$

Here, $\varepsilon_1(\omega)$ and $\varepsilon_2(\omega)$ are the real and the imaginary parts of the dielectric function obtained from the 3D supercell calculation. $L_z$ is the supercell dimension along the out-of-plane direction, $\omega$ is the photon frequency and $c$ is the speed of light in vacuum. As shown in Figures S6a and S6b, the curve shapes of the 2D polarizability are identical to those of the dielectric function presented in Figure 4a and Figure 3a of the main text, differing only in the vertical scale. The same trends are observed: a clear redshift from BeO to CdO and opposite trends in the dominant peak intensity between Groups IIA and IIB. For group IIA, the dominant peak intensity (both $\text{Re}[\alpha_{2\text{D}}(\omega)]$ and $\text{Im}[\alpha_{2\text{D}}(\omega)]$) decreases with increasing atomic number (BeO: 44.67, 87.50; MgO: 42.95, 71.89; CaO:14.67, 20.33), while in Group IIB, CdO (113.51, 192.20) exhibits a stronger peak than ZnO (37.28, 61.21). Furthermore, the optical absorbance $A(\omega)$ in Figure S6c exhibits an identical spectral profile to the absorption coefficient shown in Figure 3c, with differences arising only from the scale of the ordinate. The consistency between the intrinsic 2D and the 3D-derived quantities confirms our comparative analysis.

**References**


1. Abdullah, N.R.; Abdullah, B.J.; Rshid, H.O.; Tang, C.-S.; Manolescu, A.; Gudmundsson, V. Enhanced electronic and optical responses of nitrogen- or boron-doped BeO monolayer: First principle computation. *Superlattices Microstruct.* **2022,** *162*, 107102. https://doi.org/10.1016/j.spmi.2021.107102
2. Hoat, D.M.; Van On, V.; Nguyen, D.K.; Naseri, M.; Ponce-Perez, R.; Vu, T.V.; Rivas-Silva, J.F.; Hieu, N.N.; Cocoletzi, G.H. Structural, electronic and optical properties of pristine and functionalized MgO monolayers: a first principles study. *RSC Adv.* **2020,** *10*, 40411-40420. https://doi.org/10.1039/d0ra05030j
3. Hoat, D.M.; Naseri, M.; Rivas-Silva, J.F.; Cocoletzi, G.H. Electronic, optical and thermoelectric properties of CaO mono- and bi-layers: Theoretical comparative investigation. *Optik* **2020,** *218*, 165115. https://doi.org/10.1016/j.ijleo.2020.165115
4. Guan, Y.; Yao, H.; Zhan, H.; Wang, H.; Zhou, Y.; Kang, J. Optoelectronic properties and strain regulation of the 2D $WS_2$/ZnO van der Waals heterostructure. *RSC Adv.* **2021,** *11*, 14085-14092. https://doi.org/10.1039/d1ra01877a
5. Hoat, D.M.; Naseri, M.; Vu, T.V.; Rivas-Silva, J.F.; Hieu, N.N.; Cocoletzi, G.H. Structural, electronic and optical properties of CdO monolayer and bilayers: Stacking effect investigations. *Superlattices Microstruct.* **2020,** *145*, 106644. https://doi.org/10.1016/j.spmi.2020.106644
6. Wakhare, S.Y.; Deshpande, M.D. Structural, electronic and optical properties of metalloid element (B, Si, Ge, As, Sb, and Te) doped g-ZnO monolayer: A DFT study. *J Mol Graph Model* **2020,** *101*, 107753. https://doi.org/10.1016/j.jmgm.2020.107753
7. Chaudhuri, S.; Das, A.K.; Das, G.P.; Dev, B.N. Ab initio study of electronic and lattice dynamical properties of monolayer ZnO under strain. *J. Electron. Mater.* **2022,** *52*, 1633-1643. https://doi.org/10.1007/s11664-022-09938-4
